# Editorial: Radicalization Among Adolescents

**DOI:** 10.3389/fpsyt.2022.917557

**Published:** 2022-05-09

**Authors:** Guillaume Bronsard, Adrian Cherney, Floris Vermeulen

**Affiliations:** ^1^Service de Psychiatrie de l'Enfant et de l'Adolescent, Université de Bretagne Occidentale, Brest, France; ^2^School of Social Science, The University of Queensland, Brisbane, QLD, Australia; ^3^Department of Political Science, University of Amsterdam, Amsterdam, Netherlands

**Keywords:** radicalization, adolescents, Europe, Canada, Australia, violent extremism

Youth radicalization is an ongoing and growing challenge worldwide (1, 2). Adolescence is a turbulent time for young people, which can generate psychologically vulnerabilities that lead young people to be attracted to and recruited into different violent extremist ideologies and groups (3, 4). However, much of what is known about radicalization and violent extremism has been derived from the study of adult terrorists. The question remains as to whether findings from this existing research can be applied to youth radicalization. One issue scholars agree on is that adolescence is accompanied by a search for identity, which has consistently been identified as a key driver for violent radicalization (1, 3, 5). Though the role that psychopathology plays in the radicalization process of adolescents has not yet been studied sufficiently, nor how programs can address specific vulnerabilities.

In the Frontiers for Psychiatry Research Topic: *Radicalization Among Adolescents* authors from various countries address this issue and illustrate how youth are disproportionately represented in radicalized networks across different continents. Now more than ever it is essential scholars and policymakers attempt to better understand the underlying mechanisms and factors driving youth radicalization and formulate effective policies and interventions. In addition to addressing this priority, this Research Topic also considers the role that psychopathological factors play in individual processes of youth radicalization and examines how young people who hold extremist views and experience psychological vulnerabilities, can be best supported by professionals.

A number of articles in this Research Topic, explore various underlying mechanisms of youth radicalization ranging from the influence of personal uncertainly and negative outlooks about life (Miconi et al.); to social networks and psychological manipulation (González et al.), mental health and trauma (Rolling et al.), and psychopathology (Duits et al.). Several papers show that that the relationship between psychopathology and violent extremism is not straightforward or one-dimensional and that it interacts with other factors. One of several articles by French scholars illustrate that psychological trauma can create vulnerabilities to radicalization amongst youth. In such cases treatments targeting psychological trauma may be one way to prevent violent radicalization.

Among practitioners there can be a lack of specialist knowledge with regards to psychopathology and extremism among young people. A demonstrated by a Dutch paper such knowledge and expertise is not always available (Vermeulen et al.). This can increase the risk of biased and incorrect interpretations of what places youth at risk of radicalization. Prevailing public and political assumptions about the causes and manifestations of violent extremism can reinforce particular viewpoints and lead to inappropriate counseling and treatment options for radicalized youth across different ideological spectrums. How best to intervene with radicalized youth is a developing field of knowledge and experience is in its infancy. As Australian experience highlights, pluralistic, non-punitive multidisciplinary approaches can be used to address various developmental and psychosocial vulnerabilities, in addition to violent extremist risk factors amongst young offenders (Barracosa and March). These approaches should be supplemented by youth-specific practitioner expertise, and a range of violent extremism case management and risk assessment measures. In another paper by French scholars, the authors conclude that professionals must consider individual risk factors and vulnerabilities, but also need to tap into existing strengths, resilience and coping strategies that youth at risk of radicalization can possess and implement forms of psychotherapeutic support for this group (Bronsard et al.).

This Research Topic shows that research from different fields of study including psychology, sociology, psychiatry, educational science, political science, criminology, child and adolescent psychiatry can contribute to holistic understandings about violent radicalization amongst young people. The papers in this Research Topic come from a diverse range of countries (Canada, France, Spain, Australia, and the Netherlands) and all raise important questions about youth radicalize and what can be done about it. This includes whether young people involved in violent extremism differ in their risks and vulnerabilities compared to adults who have radicalized or other “ordinary” delinquents? Does this therefore require different forms of tailored treatment? Findings from papers in this Research Topic are derived from cohort studies and individual case studies and all draw attention to overlapping and divergent factors and processes. While the evaluation of youth specific deradicalization programs is lacking [(5), Barracosa and March], practitioner experience in the field of juvenile justice and mental health can provide important lessons and needs to be collated and disseminated to help inform policy and practice. While the problem of youth radicalization has been raised as a serious problem in need of a solution by politicians, the media and security agencies, it is essential responses are evidence-based. The collection of papers in this Research Topic provide an important contribution to emerging knowledge and the evidence-base on adolescent radicalization.

## Author Contributions

All authors listed have made a substantial, direct, and intellectual contribution to the work and approved it for publication.

## Conflict of Interest

The authors declare that the research was conducted in the absence of any commercial or financial relationships that could be construed as a potential conflict of interest.

## Publisher's Note

All claims expressed in this article are solely those of the authors and do not necessarily represent those of their affiliated organizations, or those of the publisher, the editors and the reviewers. Any product that may be evaluated in this article, or claim that may be made by its manufacturer, is not guaranteed or endorsed by the publisher.
